# Asthma under control is inversely related with erosive esophagitis among healthy adults

**DOI:** 10.1371/journal.pone.0210490

**Published:** 2019-01-07

**Authors:** Joo Hyun Lim, Dong Ho Lee, So Hee Lee, Joo Sung Kim, Hyun Chae Jung, Sang-Heon Cho

**Affiliations:** 1 Seoul National University Hospital, Healthcare System Gangnam Center, Healthcare Research Institute, Seoul, Korea; 2 Department of Internal Medicine, Seoul National University Bundang Hospital, Seongnam, Korea; 3 Department of Internal Medicine and Liver Research Institute, Seoul National University College of Medicine, Seoul, Korea; 4 Department of Internal Medicine Seoul National University College of Medicine, Seoul, Korea; Nagoya University, JAPAN

## Abstract

**Background:**

Some recent studies suggested that reflux esophagitis is positively correlated with asthma. However, there are debates on this issue. The aim of this study is to clarify the true association between reflux esophagitis and asthma in a large population.

**Methods:**

Medical records of subjects who received health surveillance checkup between January 2005 and December 2011 were reviewed. Their endoscopic findings, medical history, body mass index, and smoking history were analyzed. Erosive esophagitis was defined as endoscopically detected mucosal break at the Z-line, irrespective of reflux symptom. Information about asthma history was obtained from their questionnaires and medical records.

**Results:**

Out of the total 15,999 patients, 986 had erosive esophagitis and 376 had asthma. In this population, erosive esophagitis was inversely related with asthma in univariable analysis (OR, 0.586; 95% CI, 0.342–1.003, *p* = 0.049). In multivariable analysis, asthma was demonstrated as an independent negative risk factor for erosive esophagitis (OR, 0.472; 95% CI, 0.257–0.869, *p* = 0.016), under adjustment with age (OR, 1.000; 95% CI, 0.994–1.006, *p* = 0.977), male sex (OR, 2.092; 95% CI, 1.683–2.601, *p* < 0.001), body mass index (OR, 1.115; 95% CI, 1.090–1.141, *p* < 0.001), smoking (OR, 1.584; 95% CI, 1.318–1.902, *p* < 0.001), and urban residence (OR, 1.363; 95% CI, 1.149–1.616, *p* < 0.001). Likewise, erosive esophagitis was shown to be an independent negative risk factor for asthma (OR, 0.558; 95% CI, 0.324–0.960, *p* = 0.035) under adjustment with age (OR, 1.025; 95% CI, 1.015–1.034, *p* <0.001), male sex (OR, 0.861; 95% CI, 0.691–1.074, *p* = 0.185), and body mass index (OR, 1.067; 95% CI, 1.030–1.106, *p* < 0.001) in multivariable analysis.

**Conclusions:**

Contrary to previous studies, this large scale data showed inverse association between erosive esophagitis and asthma. Further studies investigating the clear mechanism of this phenomenon are warranted.

## Introduction

Asthma is one of the major chronic immunologic diseases, from which approximately 300 million people are suffering worldwide and the number is continuously growing [1]. Gastroesophageal reflux disease is another widespread and increasing disorder, in which gastric acid abnormally rise over the lower esophageal sphincter and almost one third of the general population experience reflux event at least once a month [2], and the prevalence of gastroesophageal reflux disease is reported to be approximately 10–20% in Western countries and 5% in Asia [3]. The reason for recent increase of asthma is assumed to be the hygiene improvement which induces impaired immunity and the increase of gastroesophageal reflux might have been caused by the contemporary late night diet pattern and various food and drugs loosening low esophageal sphincter. Recently, there have been many reports supporting positive association between asthma and gastroesophageal reflux disease [4–9]. There have been two hypotheses about this association: one is the reflux hypothesis, which suggests that the refluxed gastric acid directly affects airway responsibility, and the other is the reflex hypothesis, which claims that the lower pH in distal esophagus stimulates vagal receptor, causing bronchospasm [10]. Nevertheless, there are still debates on this issue, because most of the evidences failed to show the causality or the timing of each event. Also, most of the studies only showed symptomatic association but the objective findings such as pulmonary function revealed conflicting results. Furthermore, the facts that many researchers studied only severe, refractory asthmatic cases excluding patients with relatively mild symptoms and that the positive association was only revealed in symptomatic reflux patients make this relationship less reliable. Therefore this research was designed to evaluate the true relationship between asthma and gastroesophageal reflux disease in large general population.

## Materials and methods

### Study population

Healthy adult subjects ≥18 years old who had undergone health checkups including upper endoscopy at the Seoul National University Hospital Healthcare System Gangnam Center in the year of 2011 were enrolled in this study. Amongst them, a questionnaire containing following factors was conducted by a trained interviewer: residence, smoking history, current medication, and history of asthma diagnosis. Body mass index (BMI) was calculated using each subject’s height and body weight measured that day. Smoking status was divided into two categories including positive (current or past) and negative (never). Eosinophil count was obtained from complete blood count test. For asthmatic patients, pulmonary function test (PFT) result was analyzed. The data of this prospectively collected cohort were retrospectively reviewed.

The Institutional Review Board of Seoul National University Hospital approved this study (IRB No. H-1612-053-813). This study complied with the Declaration of Helsinki. Informed consent was waived because of the retrospective nature of this study and the data were analyzed anonymously.

### History of asthma diagnosis

To confirm each patient’s history of asthma diagnosis, we reviewed the answers to the questionnaires and his/her previous medical records. Regardless of current asthma medication, those with asthma diagnosis by a physician were considered to have history of asthma. To enhance the reliability of the answers, their previous answers taken during the previous regular health checkups were all reviewed when available. When the previous multiple answers about asthma diagnosis were inconsistent, their medical records and/or current medication were reviewed. In such cases, those who had definite records about asthma diagnosis or current medication including bronchodilator for asthma were taken to have true asthma history.

### Status of erosive esophagitis

In this study, endoscopically confirmed erosive esophagitis was defined as reflux esophagitis. Erosive esophagitis was confirmed by endoscopic evaluation based on Los Angeles classification (LA-A: one or more mucosal breaks <5mm in maximal length; LA-B: one or more mucosal breaks >5mm, but without continuity across mucosal folds; LA-C: mucosal beaks continuous between >2 mucosal folds, but involving less than 75% of the esophageal circumference; grade D: mucosal breaks involving more than 75% of esophageal circumference) [11]. In this study those with LA-A or higher grade of erosions were counted to have erosive esophagitis.

### Statistical analysis

Chi-square test or Fisher’s exact test was used to evaluate categorical variables. Fisher’s exact test was used only when more than 20% of expected frequencies were 5 or less. Student’s *t-*test was used to analyze continuous variables. Variables with *p-*values < .20 were included in multivariable analysis. For multivariable analysis, a binary logistic regression model was used. In multivariable analysis, variables with *p-*values < .05 were considered as independently related with the outcome variable. All results were presented without multiple testing. All statistical analyses were performed using the Statistical Package of the Social Sciences, ver. 23 (SPSS Inc., Chicago, IL, USA).

## Results

**Fig 1:**
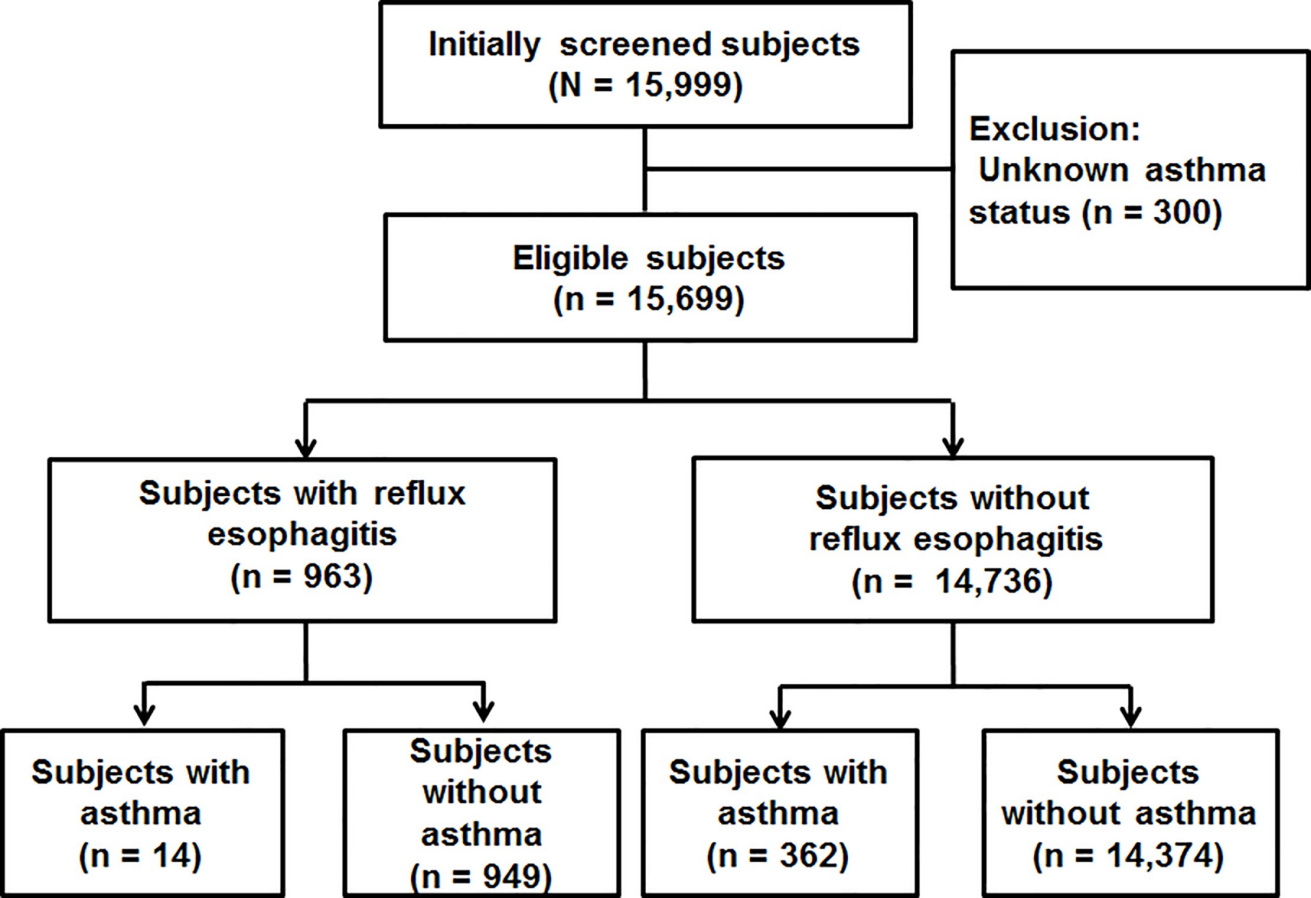
Schematic flowchart for subjects enrolled in this study. A total of 15,699 individuals who underwent health checkups including upper gastrointestinal endoscopy were enrolled in this study.

Initially, 15,999 subjects who underwent health surveillance checkup including upper endoscopy were screened (Fig 1) and 300 were excluded because of uncertain or no answer to the question about history of asthma diagnosis. Therefore, the remaining 15,699 were confirmed to be eligible. Amongst them, 963 (6.1%) were found to have erosive esophagitis by endoscopy and 376 (2.4%) answered to have asthma. Among those with asthma, 14 (3.7%) had erosive esophagitis, while among those with erosive esophagitis, 14 (1.5%) had asthma. For asthmatics, we analyzed its severity according to their forced expiratory volume at 1 second (FEV1) measured during the PFT. Those who had FEV1 ≥80% predicted were counted as mild asthmatics, those with FEV1 <60% predicted were taken as severe asthmatics, and the remaining were defined as moderate asthmatics. In this analysis, 313 (83.2%) were in mild status, 44 (11.7%) were in moderate status, and only 19 (5.1%) were in severe status. Among those with erosive esophagitis, 761 (79.0%) were in LA-A, 175 (18.2%) were in LA-B, and only 27 (2.8%) were in LA-C.

### Baseline characteristics

**Fig 2:**
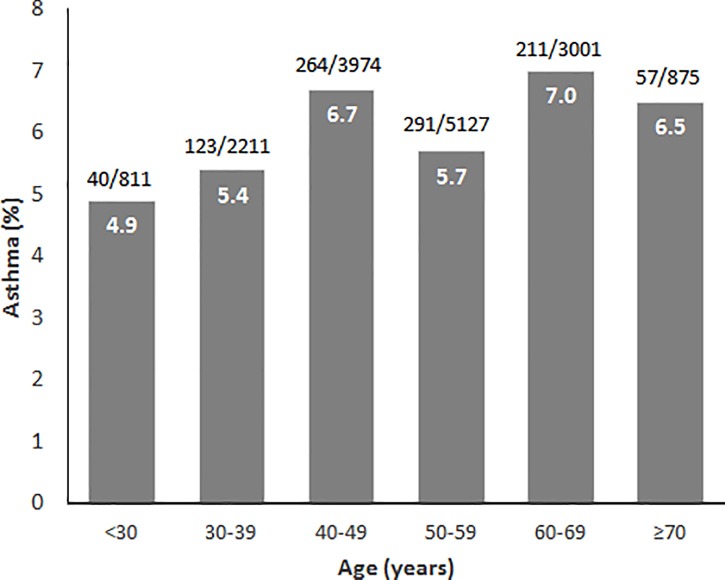
Prevalence of erosive esophagitis according to age. Prevalence of erosive esophagitis showed slightly increasing trend along with age.

**Fig 3:**
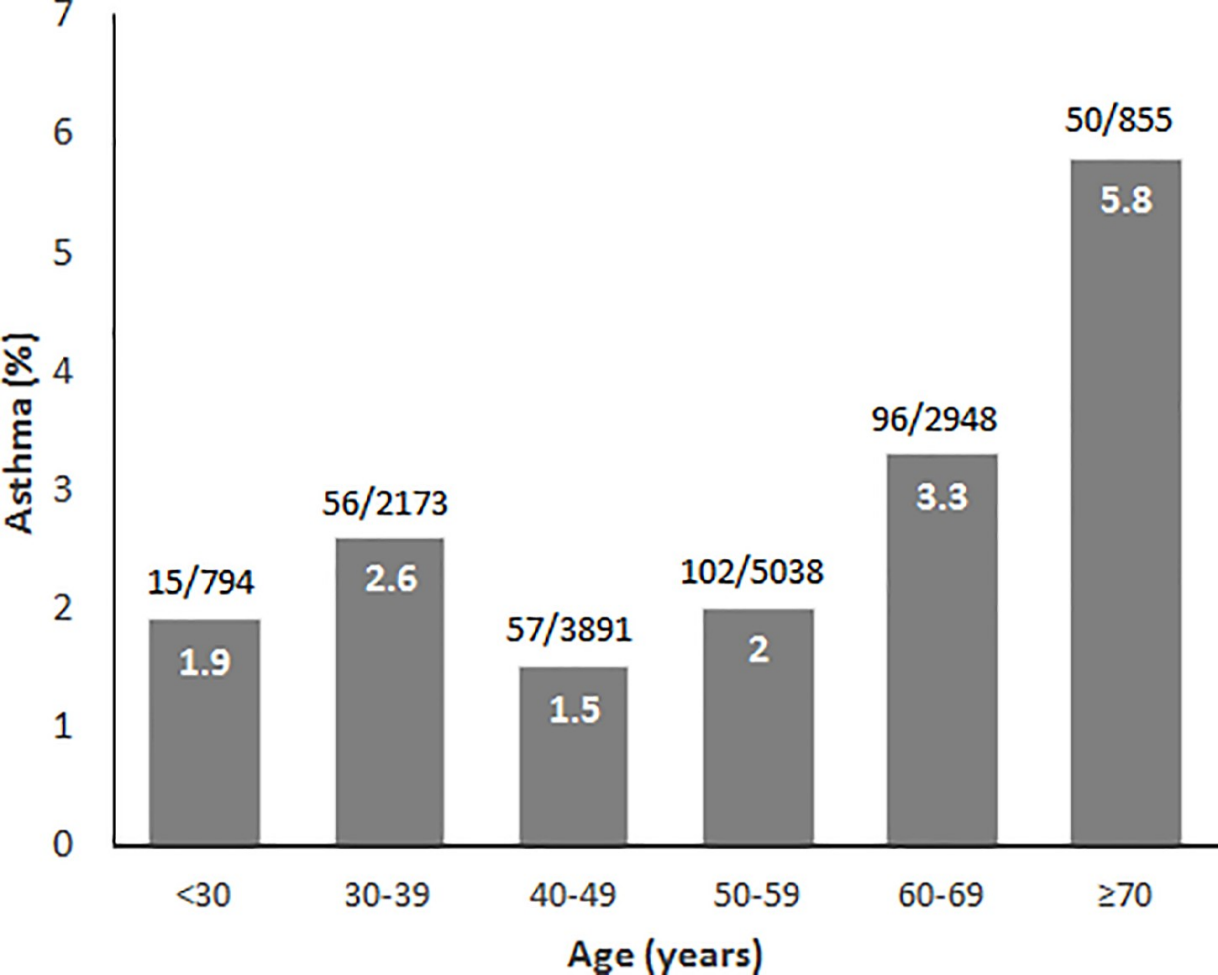
Prevalence of asthma according to age. Asthma prevalence according to age shifted before and after the age of 50. It tended to slightly decrease by age in those under their fifties and since then dramatically increased by age.

The mean age of the total subjects was 50.7 and male proportion was about 53% (Table 1). Mean BMI was 23.3. About half were current or ex-smokers and 76% were living in urban area. Those who had erosive esophagitis were more likely to be male, with higher BMI, with smoking history, and living in urban area than those without erosive esophagitis. According to asthma status, those with asthma were older and had higher BMI than those without asthma. In terms of age distribution, erosive esophagitis showed slightly increasing pattern as the age increases, although it failed to demonstrate statistical significance (OR, 1.005; 95% CI, 1.000–1.010, *p* = 0.067, Fig 2). On the other hand, asthma prevalence revealed increasing pattern as the age increases with statistical significance (OR, 1.026; 95% CI, 1.017–1.035, *p* < 0.001, Fig 3). However, among those younger than 50 years, the pattern was inverse (OR, 0.974; 95% CI, 0.952–0.996, *p* = 0.019), and after the age of 50 the slope shifted toward positive (OR, 1.063; 95% CI, 1.046–1.080, *p* < 0.001).

**Table 1:** Baseline demographic characteristics.

	Erosive esophagitis			Asthma			Overall (n = 15699)
	Positive (n = 963)	Negative (n = 14736)	*p*-value	Positive (n = 376)	Negative (n = 15323)	*p*-value	
**Age, mean ± SD (years)**	51.4 ± 11.8	50.7 ± 12.2	0.07	54.3 ± 14.1	50.6 ± 12.1	<0.01	50.7 ± 12.2
**Male, n (%)**	769 (79.9)	7606 (51.6)	<0.01	201 (53.5)	8174 (53.3)	0.97	8375 (53.3)
**BMI, mean ± SD**	24.8 ± 3.1	23.2 ± 3.1	<0.01	23.9 ± 3.3	23.3 ± 3.1	<0.01	23.3 ± 3.1
Smoking*, n (%)			<0.01			0.59	
**Nonsmoker**	280/917 (30.5)	7728/13828 (55.9)		183/346 (52.9)	7825/14399 (54.3)		8008/14745 (44.3)
**Current/past smoker**	637/917 (69.5)	6100/13828 (44.1)		163/346 (47.1)	6574/14399 (45.7)		6737/14745 (45.7)
Residence*, n (%)			0.01			0.61	
**Rural**	193 (20.0)	3517 (23.9)		93 (24.7)	3617 (23.6)		3710 (23.6)
**Urban**	770 (80.0)	11219 (76.1)		283 (75.3)	11706 (76.4)		11989 (76.4)

SD, standard deviation; BMI, body mass index

* The denominator is less than the total number, because of missing data.

### Factors related to erosive esophagitis

Univariable analysis for erosive esophagitis showed that asthma (OR, 0.586; 95% CI, 0.342–1.003, *p* = 0.049), male sex (OR, 3.716; 95% CI, 3.164–4.364, *p* < 0.001), BMI (OR, 1.170; 95% CI, 1.147–1.194, *p* < 0.001), smoking (OR 2.882; 2.494–3.330, *p* < 0.001), and urban residence (OR, 1.251; 95% CI, 1.063–1.471, *p* = 0.007) were related with erosive esophagitis (Table 2). Amongst them, asthma was inversely related with erosive esophagitis. In multivariable analysis, asthma (OR, 0.472; 95% CI, 0.257–0.869, *p* = 0.016), male sex (OR, 2.092; 95% CI, 1.683–2.601, *p* < 0.001), BMI (OR, 1.115; 95% CI, 1.090–1.141, *p* < 0.001), smoking (OR, 1.584; 95% CI, 1.318–1.902, *p* < 0.001), and urban residence (OR, 1.363; 95% CI, 1.149–1.616, *p* < 0.001) were revealed to be independently associated with erosive esophagitis. Again, asthma was inversely related with erosive esophagitis.

**Table 2:** Univariable and multivariable analysis for erosive esophagitis and asthma.

	Erosive esophagitis				Asthma			
	Univariable OR (95% CI)	Univariable *p-*value	Multivariable OR (95% CI)	Multivariable *p*-value	Univariable OR (95% CI)	Univariable *p*-value	Multivariable OR (95% CI)	Multivariable *p*-value
**Age**	1.005 (1.000–1.010)	0.067	1.000 (0.994–1.006)	0.977	1.026 (1.017–1.035)	<0.001	1.025 (1.015–1.034)	<0.001
**Male**	3.716 (3.164–4.364)	<0.001	2.092 (1.683–2.601)	<0.001	1.005 (0.818–1.233)	0.965	0.861 (0.691–1.074)	0.185
**BMI**	1.170 (1.147–1.194)	<0.001	1.115 (1.090–1.141)	<0.001	1.066 (1.032–1.101)	<0.001	1.067 (1.030–1.106)	<0.001
**Smoking**	2.882 (2.494–3.330)	<0.001	1.584 (1.318–1.902)	<0.001	1.060 (0.856–1.313)	0.592	-	-
**Urban residence**	1.251 (1.063–1.471)	0.007	1.363 (1.149–1.616)	<0.001	0.940 (0.742–1.192)	0.611	-	-
**Asthma**	0.586 (0.342–1.003)	0.049	0.472 (0.257–0.869)	0.016	NA	NA	NA	NA
**Erosive esophagitis**	NA	NA	NA	NA	0.586 (0.342–1.003)	0.049	0.558 (0.324–0.960)	0.035

OR, odds ratio; CI, confidence interval; BMI, body mass index; NA, not applicable

### Factors related to asthma

In univariable analysis for asthma, erosive esophagitis (OR, 0.586; 95% CI, 0.342–1.003, *p* = 0.049), age (OR, 1.026; 95% CI, 1.017–1.035, *p* < 0.001) and BMI (OR, 1.066; 95% CI, 1.032–1.101, *p* < 0.001) were shown to be related with asthma (Table 2). Among them, erosive esophagitis was inversely related with asthma. Multivariable analysis showed erosive esophagitis (OR, 0.558; 95% CI, 0.324–0.960, *p* = 0.035), age (OR, 1.025; 95% CI, 1.015–1.034, *p* < 0.001), and BMI (OR, 1.067; 95% CI, 1.030–1.106, *p* < 0.001) are independently associated with asthma. Here again, erosive esophagitis was a negative risk factor for asthma.

### Subgroup analysis according to age group

As the asthma prevalence pattern according to age shifted at the age of 50, we performed subgroup analysis according to age groups. Among those younger than 50 years, asthma was not shown to be significantly associated with erosive esophagitis in multivariable analysis (OR, 0.557; 95% CI, 0.223–1.387, *p* = 0.209, Table 3), while male sex (OR, 2.052; 95% CI, 1.489–1.828, *p* < 0.001), BMI (OR, 1.123; 95% CI, 1.088–1.160, *p* < 0.001), smoking (OR, 1.636; 95% CI, 1.256–2.131, *p* < 0.001), and urban residence (OR, 1.393; 95% CI, 1.060–1.829, *p* = 0.017) were all shown to be independent risk factors for erosive esophagitis. On the other hand, among those over 50 years old, asthma was shown to be an independent negative risk factor for erosive esophagitis (OR, 0.418; 95% CI, 0.184–0.952, *p* = 0.038), and male sex (OR, 2.100; 95% CI, 1.554–2.838, *p* < 0.001), BMI (OR, 1.105; 95% CI, 1.068–1.142, *p* < 0.001), smoking (OR, 1.545; 95% CI, 1.195–1.998, *p* = 0.001), and urban residence (OR, 1.340; 95% CI, 1.076–1.668, *p* = 0.009) were all independent risk factors for erosive esophagitis (Table 3).

**Table 3:** Subgroup analysis according to age for erosive esophagitis and asthma.

	Erosive esophagitis				Asthma			
	<50 years old (n = 6858)		≥50 years old (n = 8841)		<50 years old (n = 6858)		≥50 years old (n = 8841)	
	Univariable OR (95% CI)	Multivariable OR (95% CI)	Univariable OR (95% CI)	Multivariable OR (95% CI)	Univariable OR (95% CI)	Multivariable OR (95% CI)	Univariable OR (95% CI)	Multivariable OR (95% CI)
**Age**	**1.020 (1.006–1.035)**	1.006 (0.991–1.022)	1.007 (0.995–1.020)	1.004 (0.991–1.018)	**0.974 (0.952–0.996)**	**0.968 (0.945–0.991)**	**1.063 (1.046–1.080)**	**1.063 (1.045–1.082)**
**Male**	**4.440 (3.467–5.687)**	**2.052 (1.489–1.828)**	**3.239 (2.622–4.002)**	**2.100 (1.554–2.838)**	1.320 (0.927–1.879)	1.066 (0.642–1.770)	0.843 (0.655–1.085)	**0.576 (0.383–0.867)**
**BMI**	**1.190 (1.159–1.222)**	**1.123 (1.088–1.160)**	**1.148 (1.114–1.183)**	**1.105 (1.068–1.142)**	1.037 (0.986–1.090)	1.036 (0.976–1.100)	**1.078 (1.031–1.127)**	**1.080 (1.030–1.133)**
**Smoking**	**3.078 (2.474–3.830)**	**1.636 (1.256–2.131)**	**2.727 (2.257–3.318)**	**1.545 (1.195–1.998)**	**1.483 (1.035–2.125)**	1.483 (0.947–2.324)	0.870 (0.665–1.137)	1.245 (0.826–1.877)
**Urban residence**	1.249 (0.961–1.624)	**1.393 (1.060–1.829)**	**1.257 (1.022–1.546)**	**1.340 (1.076–1.668)**	1.224 (0.770–1.944)	1.122 (0.701–1.795)	0.886 (0.670–1.172)	0.807 (0.601–1.083)
**Asthma**	0.623 (0.254–1.533)	0.557 (0.223–1.387)	0.565 (0.289–1.105)	**0.418 (0.184–0.952)**	NA	NA	NA	NA
**Erosive esophagitis**	NA	NA	NA	NA	0.623 (0.254–1.533)	0.554 (0.223–1.380)	0.565 (0.289–1.105)	**0.414 (0.182–0.941)**

OR, odds ratio; CI, confidence interval; BMI, body mass index; NA, not applicable. Bold style indicates statistical significance.

As for asthma, among those younger than 50 years, erosive esophagitis was not independently related with asthma (OR, 0.554; 95% CI, 0.223–1.380, *p* = 0.205), on the meanwhile, age was a negative risk factor for asthma (OR, 0.968; 95% CI, 0.945–0.991, *p* = 0.007), in multivariable analysis (Table 3). Whereas, among those over 50 years old, erosive esophagitis (OR, 0.414; 95% CI, 0.182–0.941, *p* = 0.035) and male sex (OR, 0.576; 95% CI, 0.383–0.867, *p* = 0.008) were shown to be negative risk factors for asthma, while age (OR, 1.063; 95% CI, 1.045–1.082, *p* < 0.001), and BMI (OR, 1.080; 95% CI, 1.030–1.133, *p* = 0.001) were shown to be positively associated with asthma (Table 3).

### Eosinophilia among asthmatics and erosive esophagitis patients

Among asthmatics, eosinophilia defined as eosinophil count >500/μL was found more common than none asthmatics (OR, 2.396; 95% CI, 1.876–3.061, *p* < 0.001). Among those with erosive esophagitis, eosinophilia was also more frequent than among those without erosive esophagitis (OR, 1.259; 95% CI, 1.041–1.522, *p* = 0.017). The eosinophil count was not greater among those with both erosive esophagitis and asthma compared with that of those with erosive esophagitis without asthma (*p* = 0.745). Also, amongst asthmatics, eosinophil count of those with erosive esophagitis was not greater than that of those without erosive esophagitis (*p* = 0.638).

## Discussion

This study revealed inverse relationship between asthma and erosive esophagitis among healthy adult population. This inverse relationship was especially prominent among those older than 50 years. And it was also true under adjustment with other associated factors, convincing that the negative relationship is robust.

So far asthma and gastroesophageal reflux disease were believed to have positive association. In previous reports, the prevalence of gastroesophageal reflux disease among asthmatics was reported to be about 30–80% [12–18], which is much higher than that of general population of 10–17% [19–21]. Likewise, the prevalence of asthma in those with gastroesophageal reflux (4.6%) was reported to be higher than that in general population (3.9%) in a systematic review [22]. According to the traditional concept, gastroesophageal reflux can cause airway hypersensitivity by direct effect of acid and indirect effect of neural reflex, and also asthma itself can worsen the reflux through intrathoracic negative pressure made by cough and drug-induced lowering of lower esophageal sphincter pressure.

However, our result was totally different from previous notions. It is complicated to explain the reason; however, we could assume that the positive relationship might only be true among those with moderate to severe symptoms. The previous researches were performed among patients who were already diagnosed for one of the diseases. Most patients with mild gastroesophageal reflux do not know about their condition or do not see doctors, relying on over-the-counter antacids for symptom relief and usually take years to seek medical help [23]. Therefore, those in early stages could not be counted in the general statistics. Furthermore, those who have been diagnosed with one disease have higher chance to be diagnosed with another because they visit clinics regularly, having more chances to complain of any discomfort to doctors. As our study included population that went through health checkups, we had opportunity to avoid such selection bias because of having most patients in the early stages, which might have induced the difference. Another possible explanation would be that erosive esophagitis does not represent all the gastroesophageal reflux. According to literatures, only 20–40% of patients with gastroesophageal reflux disease show erosive esophagitis [24–26] and the severity of erosion does not match that of the reflux symptom. A previous study has shown that non-erosive reflux disease patients had more frequent visits to the emergency room and longer stays in hospitals than erosive reflux disease patients [27]. Furthermore, there is a diagnostic overlap between eosinophilic esophagitis and gastroesophageal reflux disease [28]. In this study, about 14% of patients with erosive esophagitis had eosinophilia, which was significantly higher than that in those without erosive esophagitis. Therefore it might be inappropriate to say that gastroesophageal reflux is inversely related with asthma with our result. However, at least it is a significant finding that erosive esophagitis amongst healthy adults was inversely related with asthma.

Then how could erosive esophagitis be negatively associated with asthma? The reason for why is unclear, however, one of the possible explanation would be that there might be two different groups of people with gastroesophageal reflux; highly sensitive and insensitive groups. A previous systematic review revealed that medication for gastroesophageal reflux disease relieved asthma symptoms and reduced asthma medication in about 60% of patients [29]. Another study showed that using proton pump inhibitor in asthmatics with gastroesophageal reflux disease could reduce asthma symptoms mostly among those with frequent reflux symptom or massive proximal esophageal acid reflux [30], which means some refluxes are related with asthma, however, some are not. Severe acid reflux which reaches proximal esophagus may be aspirated into upper airway inducing airway hypersensitivity, while even lower level of acid reflux which only reaches distal esophagus is known to be able to stimulate vagus nerve endings to produce bronchospasm [31]. However, the fact that not all of the patients with acid reflux develop asthma is important and there must be sensitive a population and an insensitive one. In this research, as the study population was health checkup individuals there was no one having severe erosive esophagitis with LA-D and there was only small number of LA-C patients. Also, people with severe reflux symptoms would seek clinics rather than surveillance endoscopy. Thus patients with erosive esophagitis in this study might be composed of those with long-standing distal esophageal acid without severe symptoms, which means most of them might be insensitive population, not showing positive association between erosive esophagitis with asthma. Then why have they shown inverse relationship in this study? It could be because of their medical treatment. In this study, we did not perform diagnostic test for asthma amongst enrolled individuals, rather we collected their previous diagnostic history of asthma, so they must have been treated for asthma for some time. Some anti-inflammatory effect of the medication might have resolved mucosal erosions in the esophagus. Recent studies have suggested that erosive esophagitis is caused by inflammatory cytokines rather than chemical injuries by acid itself [32, 33]. Another study demonstrated that leukotriene receptor antagonist could alleviate erosive esophagitis [28]. As our population had only mild esophageal erosions, significant portion of them might have fully resolved by asthma medication. Likewise, some of them might have taken proton pump inhibitors, improving possible bronchoconstriction and ultimately removing the chance to be diagnosed for asthma because of less symptomatic conditions.

In this study, we performed subgroup analysis according to age because the asthma prevalence showed changing trend before and after the age of 50. This trend was similar to the trend of asthma prevalence according to age among general population worldwide [34]. Subgroup analysis according to age showed that the inverse relationship between asthma and erosive esophagitis was only true in relatively older population. This finding is very complicated to be explained. Anyhow, the fact that vagal reflex is more sensitive in younger population may give some clue for the difference between age groups. In other words, the older age group may be mostly composed of the population insensitive to acid reflux. Also, asthma developed in younger age is more likely to be allergic, less affected by other factors, making the two diseases independent from each other. Meanwhile, that in old age is more environment-related and older patients may have longer period of medication for asthma. Another probable explanation would be that, old people usually show better compliance than young people, and this might have influenced the medication effect.

There are several limitations to this study. First of all, this study only showed the phenomenon, not evaluated the underlying mechanism. Also, because of the retrospective nature of this study, we could not thoroughly review the medication history or chronologic change of each disease status. One other limitation is that health checkup population cannot properly represent general population. However, we still believe that this healthy adult population can better represent general population than the patients visiting clinics can do, as much larger proportion of patients with these chronic diseases are having mild symptoms, not visiting clinics.

The brand new finding that erosive esophagitis in healthy population is negatively associated with asthma gives important and noble information about asthma and reflux esophagitis. It does not suggest underlying mechanism, however, suggesting that the traditional notion of close association between asthma and gastroesophageal reflux may not be true in all population. Applying asthma medication for acid reflux or applying anti-reflux treatment for asthma cannot always be the answer. Distinguishing the proper target population would be the future challenge. For this, meticulous investigation to find the true phenomenon developing in the early stage of disease would be warranted.

## Abbreviations

BMI: body mass index
OR: odds ratio
CI: confidence interval
